# p53-dependent SIRT6 expression protects Aβ42-induced DNA damage

**DOI:** 10.1038/srep25628

**Published:** 2016-05-09

**Authors:** Eun Sun Jung, Hyunjung Choi, Hyundong Song, Yu Jin Hwang, Ahbin Kim, Hoon Ryu, Inhee Mook-Jung

**Affiliations:** 1 Department of Biochemistry & Biomedical Science, Seoul National University College of Medicine, Seoul 03080, Republic of Korea; 2 Center for Neuro-Medicine, Brain Science Institute, KIST, Seoul, Korea; 3 Department of Neurology and Pathology, Boston University School of Medicine, Boston, MA, USA

## Abstract

Alzheimer’s disease (AD) is the most common type of dementia and age-related neurodegenerative disease. Elucidating the cellular changes that occur during ageing is an important step towards understanding the pathogenesis and progression of neurodegenerative disorders. *SIRT6* is a member of the mammalian sirtuin family of anti-aging genes. However, the relationship between SIRT6 and AD has not yet been elucidated. Here, we report that SIRT6 protein expression levels are reduced in the brains of both the 5XFAD AD mouse model and AD patients. Aβ42, a major component of senile plaques, decreases SIRT6 expression, and Aβ42-induced DNA damage is prevented by the overexpression of SIRT6 in HT22 mouse hippocampal neurons. Also, there is a strong negative correlation between Aβ42-induced DNA damage and p53 levels, a protein involved in DNA repair and apoptosis. In addition, upregulation of p53 protein by Nutlin-3 prevents SIRT6 reduction and DNA damage induced by Aβ42. Taken together, this study reveals that p53-dependent SIRT6 expression protects cells from Aβ42-induced DNA damage, making SIRT6 a promising new therapeutic target for the treatment of AD.

Alzheimer’s disease (AD) is a representative progressive neurodegenerative disorder and is strongly associated with ageing^1^,^2^. The most common form of AD is late-onset, generally occurring after 65 years^3^. Unfortunately, the causes of late-onset AD are poorly understood, although there are known genes that can contribute to the development of late-onset AD. Ageing is the most important risk factor for the development of neurodegenerative diseases, including AD, Parkinson’s disease (PD), amyotrophic lateral sclerosis (ALS), and cerebrovascular disease^4^. During ageing, increased production of reactive oxygen species (ROS) and decreased capacity for DNA repair potentially contribute to the accumulation of DNA damage^5^,^6^,^7^. DNA damage accumulating during the aging process increases cellular senescence and apoptotic cell death, enhancing the risk of developing age-related diseases^5^,^8^,^9^. Notably, elevation of nuclear and mitochondrial oxidative DNA damage can occur in the brains of AD patients^10^,^11^. In addition, the activity of DNA repair proteins such as DNA-dependent protein kinase (DNA-PK)^12^, DNA polymerase β (Pol β)^13^, and 8-oxoguanine DNA glycosylase (OGG1)^14^,^15^ is impaired in AD affected brains. The brains of AD patients have amyloid plaques composed primarily of aggregated amyloid-beta (Aβ) peptide. Moreover, there are two major forms of Aβ, Aβ40 and Aβ42 and Aβ42 is more toxic to neuron than Aβ40. It has also been reported that Aβ, a main component of amyloid plaque, can induce DNA damage^16^,^17^. Therefore, understanding the brain DNA repair system is essential for the development of novel therapeutic strategies for AD.

The sirtuin gene family consists of proteins, which regulate a variety of cellular processes and are broadly conserved from bacteria to humans^18^. Silent information regulator-2 (*Sir2*), the first sirtuin protein discovered, demonstrates NAD-dependent histone deacetylase^19^ and ADP-ribosyltransferase activity^20^. The mammalian sirtuin family consists of seven members (SIRT1-SIRT7) that differ in their subcellular localization^21^. Sirtuin 6 (*SIRT6*), also known as a longevity gene, is localized mainly to the nucleus. The NAD-dependent histone deacetylase SIRT6 has multiple roles related to ageing such as telomere maintenance, DNA repair, genome integrity, energy metabolism, and inflammation, which ultimately regulate life span^22^,^23^,^24^,^25^,^26^. Recently, researchers have reported that SIRT6-deficient mice have severe metabolic defects including fatal hypoglycemia and display a premature ageing-like phenotype^22^,^27^,^28^. Defects in DNA repair and glucose metabolism have been shown to cause age-related cognitive impairment in mice and humans^29^,^30^,^31^. Therefore, defects in SIRT6 levels or function may be closely linked to late-onset/age-related AD. However, the potential role of SIRT6 in AD has remained unexplored.

Here, we report that SIRT6 expression is decreased in the brains of an AD mouse model and also in AD patients. We found that Aβ42 significantly decreased SIRT6 expression, which was associated with decreased p53 levels by Aβ42. Ubiquitin-proteasome pathway regulates p53 stability through a mouse double minute 2 homolog (MDM2) which is an E3 ligase and promotes p53 degrdation^32^. Because Nutlin-3, a selective small-molecule antagonist of MDM2, disrupts p53-MDM2 interaction and increases p53 stability^33^, we also showed that activation of p53 prevents Aβ42-induced reduction of SIRT6 levels and DNA damage using Nutlin-3. Furthermore, the protective effect of Nutlin-3 on Aβ42-mediated DNA damage requires SIRT6 expression by p53. Therefore, we anticipate that SIRT6 will be a novel therapeutic target for AD.

## Results

### Decreased levels of SIRT6 in the brains of 5XFAD mice and AD patients

**Figure 1:**
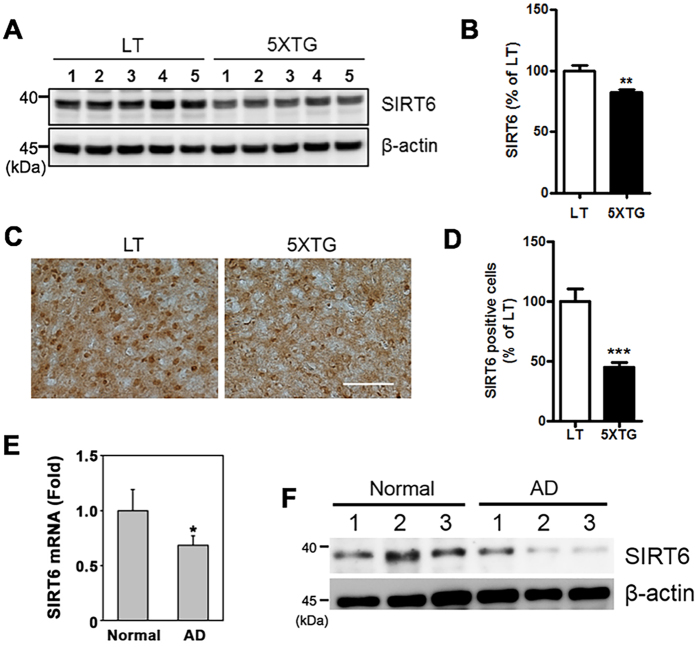
SIRT6 levels in the brains of 5XFAD mice and AD patients. SIRT6 downregulation in the brains of 5XFAD mice and AD patients. (**A**) Western blotting for SIRT6 protein levels from hippocampal tissue isolated from 6 month old 5XFAD mice (5XTG) or littermate control mice (LT). Protein expression was analyzed using total protein extract. (**B**) Quantification of SIRT6 in Fig. 1A (**P < 0.01 versus littermate control mice, unpaired t-test). (**C**) Representative DAB staining images of SIRT6 in the frontal cortex of LT or 5XTG mice (9 month old). Scale bar: 200 μm **(D**) Quantification of SIRT6 positive cells (n = 5, ***P < 0.001). (**E**) The mRNA level of SIRT6 is significantly decreased in the frontal cortex of AD patients (n = 6) compared to normal subjects (n = 6) (*p < 0.05 versus normal subjects). (**F**) The protein level of SIRT6 is reduced in the brains of AD patients compared to normal subjects. All the gels were run under same experimental conditions. Full-length images are presented in the supplementary information.

To gain insight into the function of SIRT6 in AD, we examined its expression level in the brains of 5XFAD mice. SIRT6 levels were decreased in both the hippocampus and frontal cortex of 5XFAD mice compared to those of wild-type littermates as judged by western blotting and immunohistochemistry (Fig. 1A–D). Furthermore, we also observed decreased SIRT6 mRNA expression in AD patients compared to unaffected individuals (Fig. 1E). We also found a corresponding decrease in SIRT6 protein levels in AD patients (Fig. 1F).

### Aβ42 inhibits the expression of SIRT6 and increases acetylation of H3K9 and H3K56

**Figure 2:**
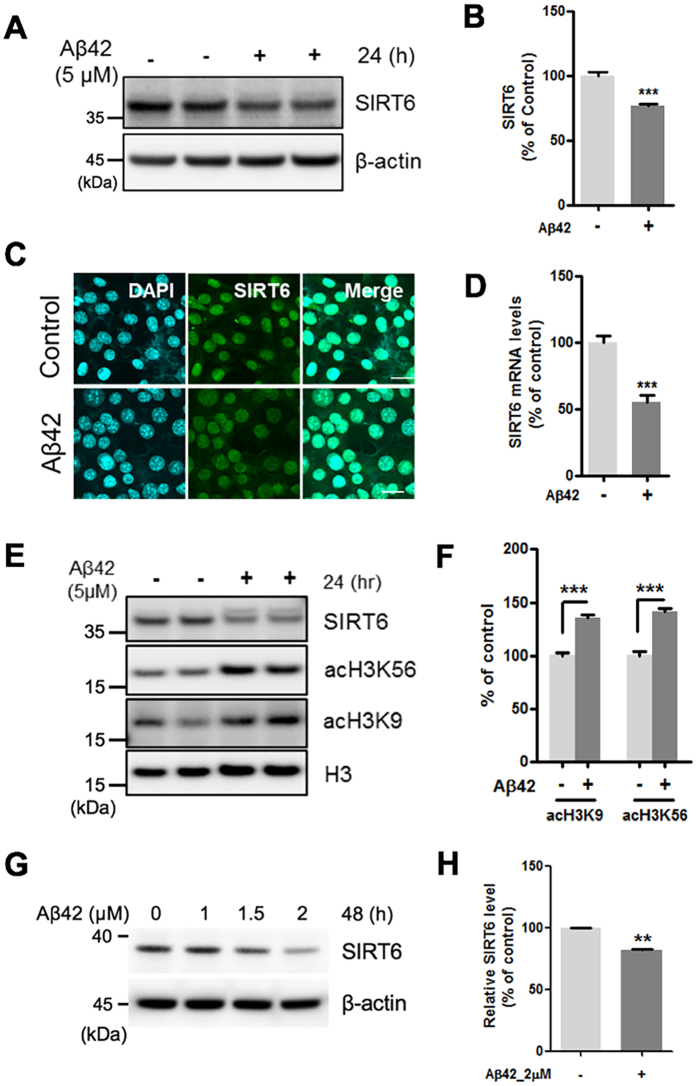
The effect of Aβ42 on the expression of SIRT6 and acetylation of H3K9 and H3K56. HT22 cells were treated with Aβ42 (5 μM) for 24 h. Aβ42 decreased both the protein and the mRNA expression of SIRT6 and increased acetylation of H3K9 and H3K56. (**A**) Western blotting for SIRT6 protein in total protein from whole cell extracts. (**B**) Quantification of SIRT6 protein (n = 5, ***P < 0.001 versus vehicle, unpaired t-test). SIRT6 level was quantified by densitometry and normalized to β-actin. (**C**) Immunocytochemistry analysis for SIRT6. HT22 cells were stained with antibodies against SIRT6 (green). DAPI (blue) was used as a nuclear marker. Scale bar: 20 μm. (**D**) The mRNA level of SIRT6 was measured by quantitative real-time PCR (n = 4, ***P < 0.001 versus vehicle group, unpaired t-test). (**E**) Western blotting for acetylation of H3K9 (acH3K9) and acH3K56. (**F**) Quantification of acH3K9 and acH3K56 (n = 4, ***P < 0.001, unpaired t-test). The levels of acH3K9 and acH3K56 were quantified by densitometry and normalized to histone H3. **(G,H**) Primary rat cortical neurons were treated with indicated concentration of Aβ42 for 48h. (**G**) Representative western blot image showing the changes in the expression of SIRT6. (**H**) Quantification of SIRT6 levels in the Aβ42-treated condition (2 μM, 48 h). n = 3, **P < 0.01 versus control, unpaired t-test. All the gels were run under same experimental conditions. Full-length images are presented in the supplementary information.

Next, to examine whether Aβ42 affects SIRT6 expression, HT22 mouse hippocampal cells were treated with Aβ42. Western blotting and immunostaining revealed a decrease in SIRT6 protein levels in HT22 cells treated with Aβ42 than that in the untreated cells (Fig. 2A–C). We observed concentration and time-dependent reduction of SIRT6 protein levels in HT22 cells exposed to Aβ42 (Supplementary Figure 1). Furthermore, Aβ42 reduced SIRT6 levels in primary mouse cortical neurons (Fig. 2G,H). In addition, SIRT6 mRNA levels in Aβ42-treated cells were downregulated than that in the untreated cells as determined by real-time PCR (Fig. 2D). We also investigated the acetylation levels of K9 and K56 on histone H3 (H3K9, H3K56) which are known SIRT6 substrates^24^,^34^,^35^. Western blotting revealed increased acetylation of H3K9 and H3K56 in cells treated with Aβ42 (Fig. 2E,F). These data indicate that Aβ42 decreases the expression of SIRT6, increasing H3K9 and H3K56 acetylation.

### A JNK inhibitor decreases the Aβ42-mediated SIRT6 and p53 reduction

**Figure 3:**
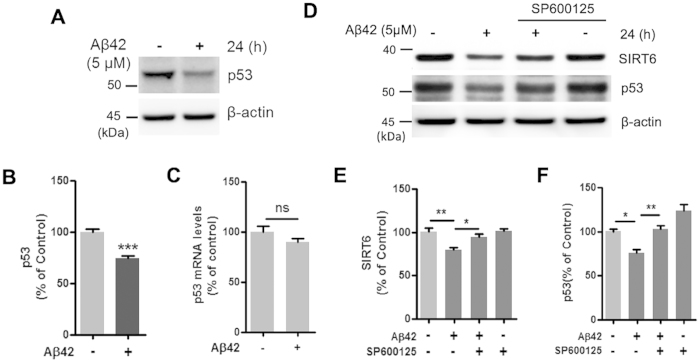
Effect of SP600125 on the Aβ42-induced reduction of SIRT6 and p53 levels. (**A–C)** HT22 cells were treated with Aβ42 (5 μM) for 24 h. Aβ42 significantly decreased p53 protein levels but did not affect p53 mRNA levels. (**A**) Representative image of western blotting for p53. Cells were harvested for immunoblotting with anti-p53 and β-actin. (**B**) Quantification of p53 protein level in (**A**) p53 level was quantified by densitometry and normalized to β-actin (n = 5, ***P < 0.001 versus vehicle, unpaired t-test). (**C**) The mRNA level of p53 was measured by quantitative real-time PCR (n = 3, ns; not significant). (**D–F**) HT22 cells were treated with Aβ42 (5 μM) for 24 h in the absence or in the presence of SP600125. SP600125 inhibited Aβ42-induced downregulation of SIRT6 and p53. (**D**) Western blotting analysis for SIRT6 and p53. (**E**) Quantification of SIRT6 protein (n = 5, *P < 0.05, **P < 0.01). (**F**) Quantification of p53 protein (n = 4, *P < 0.05, **P < 0.01). Relative expression of SIRT6 and p53 normalized to β-actin. Statistical significance was tested by one-way ANOVA followed by a Tukey’s multiple comparison test. All the gels were run under same experimental conditions. Full-length images are presented in the supplementary information.

Next, we investigated the molecular mechanism by which Aβ42 reduces SIRT6 expression. Recently, Zhang *et al*. reported that p53 directly activates the expression of SIRT6^36^. Because we found that Aβ42 reduced SIRT6 expression, we examined whether Aβ42 affects p53 expression. Interestingly, we observed a strong reduction of p53 protein in HT22 cells treated with Aβ42 (Fig. 3A,B). In contrast, p53 mRNA expression in HT22 cells was not affected by Aβ42 treatment (Fig. 3C). Several studies have indicated that the c-Jun N-terminal kinase (JNK) pathway, which includes c-Jun, can regulate SIRT6 and p53 levels^26^,^37^. In addition, Aβ activates JNK, and the JNK pathway is activated in both AD brains and the AD model mice^38^,^39^,^40^. To examine the involvement of JNK signaling in the Aβ42-induced reduction of SIRT6 and p53, HT22 cells were treated with the JNK inhibitor SP600125 and Aβ42. Co-treatment with SP600125 prevented Aβ42-induced reduction of SIRT6 and p53 levels (Fig. 3D–F), suggesting that the downregulation of SIRT6 and p53 by Aβ42 is mediated by the JNK pathway.

### Nutlin-3 prevents the reduction of SIRT6 and p53 levels by Aβ42

**Figure 4:**
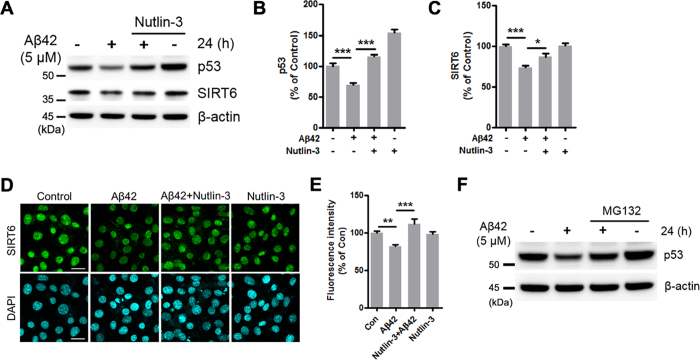
Effect of Nutlin-3 on the Aβ42-mediated reduction of SIRT6 and p53 levels. (**A–E**) HT22 cells were treated with Aβ42 (5 μM) for 24 h in the absence or in the presence of Nutlin-3, an inhibitor of the p53-mdm2 interaction. (**A**) Western blot analysis for SIRT6 and p53 protein levels. (**B,C**) Quantification of SIRT6 and p53 expression. The levels of SIRT6 and p53 were quantified by densitometry and normalized to β-actin. Statistical significance was tested by one-way ANOVA followed by a Tukey’s multiple comparison test. (n = 5, *P < 0.05, ***P < 0.001). (**D**) Immunocytochemistry analysis for SIRT6. After treatment, HT22 cells were stained with an antibody against SIRT6 (green). DAPI (blue) was used as a nuclear marker. Scale bar: 20 μm. (**E**) Quantification of the fluorescence intensity for SIRT6. The fluorescence intensity values were measured in 325 to 330 cells per group. Statistical significance was tested by one-way ANOVA followed by a Tukey’s multiple comparison test (**P < 0.01, ***P < 0.001). (**F**) Representative image of western blotting for p53 in HT22 cells treated with Aβ42, MG132, or both for 24 h. All the gels were run under same experimental conditions. Full-length images are presented in the supplementary information.

It is well known that MDM2 negatively regulates p53 stability by ubiquitination, targeting p53 for proteasome-dependent degradation^32^. Nutlin-3 is a selective antagonist of MDM2 which inhibits the p53-MDM2 interaction and consequently stabilizes p53^33^. To examine whether stabilizing p53 would rescue SIRT6 expression, HT22 cells were exposed to Aβ42 combined with Nutlin-3. Indeed, Nutlin-3 prevented the Aβ42-mediated reduction of p53 levels (Fig. 4A,B). In addition to Nutlin-3, we found that the Aβ42-induced reduction of p53 was also prevented by MG132, a proteasome inhibitor (Fig. 4F). Furthermore, we observed that the Aβ42-induced reduction of SIRT6 levels was restored by Nutlin-3 (Fig. 4A,C). The effect of Nutlin-3 on SIRT6 expression was also confirmed by immunofluorescence staining (Fig. 4D,E). These results suggest that Aβ42 decreases p53 levels through ubiquitin-proteasome system (UPS)-dependent degradation and consequently reduces SIRT6 expression.

To confirm the p53 dependent Aβ42-induced reduction of SIRT6 further, we used p53-deficient HCT116 cells (p53^−/−^ cell). In contrast to decreased SIRT6 protein levels by Aβ42 in normal cells (Figs 2, 3, 4), we observed no effect of Aβ42 on the SIRT6 from p53-deficient cells (Supplementary Figure 2a,b). Furthermore, we confirmed that Aβ42 was able to downregulate SIRT6 levels again when wild-type p53 was reintroduced into p53-deficient cells (Supplementary Figure 3). These data suggest that Aβ42 regulates SIRT6 directly through p53.

### Overexpression of SIRT6 prevents Aβ42-induced DNA damage

**Figure 5:**
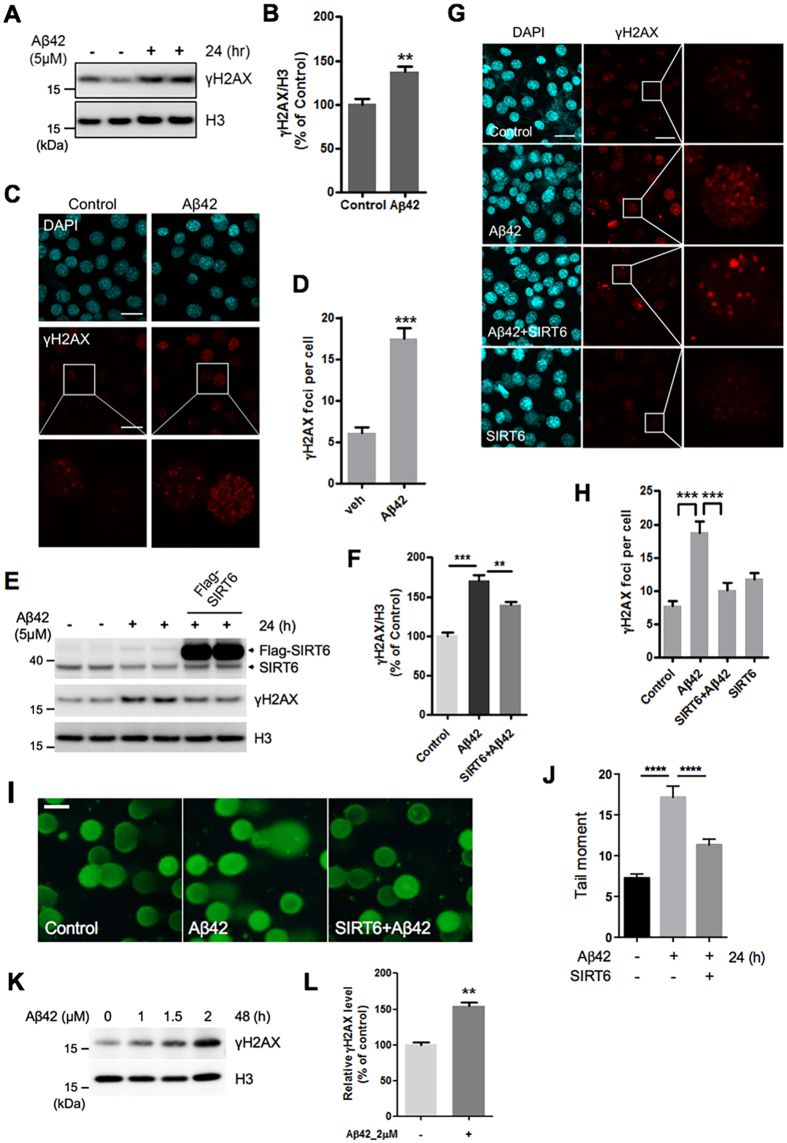
SIRT6 overexpression inhibits the DNA damage induced by Aβ42. (**A–D**) HT22 cells were treated with Aβ42 (5 μM) for 24 h. (**A,B**) Representative immunoblotting image and quantitative analysis of γH2AX (n = 5, **P < 0.01, unpaired t-test). (**C**) Representative images of γH2AX (red) stained cells. After Aβ42 treatment, HT22 cells were stained with an antibody against γH2AX (red). DAPI (blue) was used as a nuclear marker. White boxes in the images indicate the area that is enlarged and shown at the bottom. Scale bar: 20 μm. (**D**) Quantification of γH2AX foci per cell detected in (**C**). The number of γH2AX foci was counted in 75 cells per each condition. ***P < 0.001, unpaired t-test. (**E–J**) HT22 cells were transiently transfected with Flag-SIRT6 24 h before Aβ42 treatment. (**E**) The expression level of each protein was assessed by immunoblotting. Histone H3 was used as a loading control. (**F**) Quantification of γH2AX levels. n = 4, **P < 0.01, ***P < 0.001, one-way ANOVA ; Tukey’s multiple comparison test. (**G**) Representative images of γH2AX (red) stained cells. DAPI (blue) was used as a nuclear marker. Scale bar: 20 μm. (**H**) Quantification of γH2AX foci per cell detected in (**G**). The number of γH2AX foci was counted in 65 to 70 cells per each condition. ***P < 0.001, one-way ANOVA; Tukey’s multiple comparison test. (**I**) Representative alkaline comet assay image. DNA damage was assessed using an alkaline comet assay in HT22 cells. DNA was labeled with SYBR green dye. Scale bar: 100 μm. (**J**) The comet tail moments (tail DNA% × length of tail), expressed as DNA damage, were analyzed from at least 100 cells in each sample. ****P < 0.0001, one-way ANOVA ; Tukey’s multiple comparison test. (**K,L**) Primary rat cortical neurons were treated with indicated concentration of Aβ42 for 48h. (**K**) Representative western blot image showing the changes in the expression of γH2AX. **L.** Quantification of γH2AX levels in the Aβ42-treated condition (2 μM, 48 h). n = 3, **P < 0.01, unpaired t-test. All the gels were run under same experimental conditions. Full-length images are presented in the supplementary information.

Since SIRT6 is a crucial regulator of DNA repair and genome stability, we next asked whether SIRT6 could prevent Aβ42-induced DNA damage^22^,^25^. First, we examined the levels of the phosphorylation of H2AX (γH2AX), a marker of DNA damage^41^, in HT22 cells after treatment with Aβ42. The levels of γH2AX were significantly elevated in cells treated with Aβ42 (Fig. 5A,B). This result was also confirmed in primary cortical neurons (Fig. 5K,L). In addition, we detected a higher number of γH2AX foci in Aβ42-treated HT22 cells (Fig. 5C,D). To investigate the effect of SIRT6 on Aβ42-induced DNA damage, HT22 cells were transfected with a construct expressing Flag-SIRT6. We found that SIRT6 overexpression reduced γH2AX levels in cells treated with Aβ42 (Fig. 5E,F). We further confirmed the western blot data by immunofluorescence analysis of γH2AX foci in cells. Consistently, Aβ42 increased the number of γH2AX foci while SIRT6 expression reduced their frequency (Fig. 5G,H). Furthermore, we also performed comet assay to compare levels of DNA damage in single cells and observed that Aβ42-induced increase in tail moment was significantly decreased by SIRT6 overexpression (Fig. 5I,J). Together, these observations strongly indicate that SIRT6 expression rescues Aβ42-induced DNA damage.

### Nutlin-3 prevents Aβ42-induced DNA damage through SIRT6

**Figure 6:**
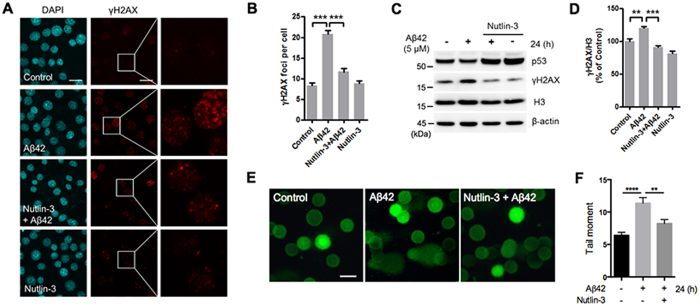
Effect of Nutlin-3 on Aβ42-induced DNA damage. HT22 cells were treated with Aβ42, Nutlin-3 or Aβ42 + Nutlin-3 for 24 h. Nutlin-3 inhibited the level of γH2AX increased by Aβ42. (**A**) Representative images of γH2AX (red) stained cells. DAPI (blue) was used as a nuclear marker. Right column is an enlargement of the boxed area in the γH2AX staining images. Scale bar: 20 μm. (**B**) Quantification of γH2AX foci per cell detected in (**A**). The number of γH2AX foci was counted in 240 cells per each condition. Statistical significance was tested by one-way ANOVA followed by a Tukey’s multiple comparison test. ***P < 0.001. (**C**) Representative image of western blots for p53 and γH2AX. (**D**) Quantification of γH2AX levels. The level of γH2AX was quantified by densitometry and normalized to histone H3. Statistical significance was tested by one-way ANOVA followed by a Tukey’s multiple comparison test (n = 4, **P < 0.01, ***P < 0.001). All the gels were run under same experimental conditions. Full-length images are presented in the supplementary information. (**E**) Representative alkaline comet assay image. DNA damage was assessed using an alkaline comet assay in HT22 cells. DNA was labeled with SYBR green dye. Scale bar: 100 μm. (**F**) The comet tail moments were analyzed from at least 100 cells in each sample using Comet Assay IV software. Statistical significance was tested by one-way ANOVA followed by a Tukey’s multiple comparison test. ****P < 0.0001, **P < 0.01.

**Figure 7:**
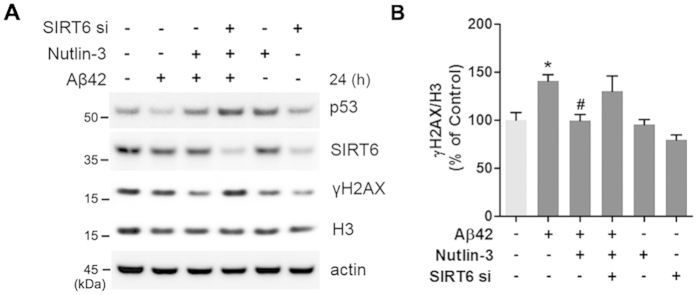
SIRT6 is required for the protective effect of Nutlin-3 on Aβ42-induced DNA damage. HT22 cells were transfected with SIRT6-specific siRNA. Twenty-four hours after transfection, cells were treated as indicated for an additional 24 h. (**A**) Western blot analysis for SIRT6, p53 and γH2AX levels. β-actin and histone H3 were used as loading control. (**B**) Quantification of γH2AX level is shown in (**A**). Statistical significance was tested by one-way ANOVA followed by a Tukey’s multiple comparison test (n = 5, *P < 0.05 versus vehicle, ^#^P < 0.05 versus Aβ42-treated groups). All the gels were run under same experimental conditions. Full-length images are presented in the supplementary information.

Because we had observed that Nutlin-3 inhibited the Aβ42-mediated reduction of SIRT6, and overexpression of SIRT6 prevented Aβ42-induced DNA damage, we examined the ability of Nutlin-3 to suppress Aβ42-induced DNA damage. We observed that Nutlin-3 relieved the Aβ42-induced increase in γH2AX foci (Fig. 6A,B). Consistent with these data, γH2AX protein levels were increased by Aβ42 but were rescued by Nutlin-3 (Fig. 6C,D). We also confirmed this protective effect of Nutlin-3 using comet assay. Nutlin-3 prevented the increased tail moment by Aβ42 (Fig. 6E,F). To explore whether this protective effect of Nutlin-3 on Aβ42-mediated DNA damage is related to p53-dependent SIRT6 expression, we determined the effect of Nultin-3 in p53-deficient HCT116 cells. Importantly, when Aβ42-induced the alterlation of SIRT6 levels did not occur in the absence of p53, Nutlin-3 had no effect on Aβ42-mediated increase in γH2AX levels (Supplementary Figure 2a,c). Furthermore, to determine whether p53 regulates Aβ42-mediated DNA damage through SIRT6, we treated HT22 cells with siRNA targeting SIRT6. The protective effect of Nutlin-3 on increased γH2AX levels by Aβ42 was abolished in cells depleted of SIRT6 (Fig. 7A,B). Although p53 was upregulated by Nutlin-3, Nutlin-3 did not protect the cells from DNA damage caused by Aβ42 when SIRT6 was downregulated by siRNA (Fig. 7). These observations suggest that p53-dependent SIRT6 expression has a critical role in preventing DNA damage promoted by Aβ42.

## Discussion

Several studies have suggested important roles of the sirtuin family for ageing-associated pathological conditions such as diabetes, neurodegeneration, inflammation, and longevity^42^,^43^,^44^,^45^. SIRT6 knockout mice have dramatically shortened life spans and display a premature ageing-like phenotype^22^. Here, we report the first study of SIRT6 expression changes in the brains of an AD model mouse and AD patients, and we reveal a protective role of SIRT6 on Aβ42-mediated DNA damage. Furthermore, we found that SIRT6 expression is regulated by p53 in response to Aβ42, which is linked to the JNK pathway.

We observed decreases of SIRT6 protein levels in the brains of both an AD model mouse and AD patients, suggesting a conserved relationship between the pathological features of AD and SIRT6 expression. Therefore, we examined whether Aβ42 regulates SIRT6 expression and we found that Aβ42 decreased SIRT6 expression (Fig. 2A). Interestingly, we found that Aβ42 treatment significantly reduced p53 levels, a tumor suppressor that plays an important role in cell-cycle control and DNA repair. mRNA and protein levels of SIRT6 were decreased by Aβ42, but p53 mRNA was not affected by this treatment. This result indicates that Aβ42 regulates SIRT6 expression at the transcription level whereas p53 is post-transcriptionally affected by Aβ42. We hypothesized that the reduction of p53 by Aβ42 was a leading cause for the decrease in SIRT6 expression, because it was reported that p53 can directly bind to the SIRT6 promoter to regulate its expression. Additionally, the JNK pathway has been shown to be closely related to AD^38^,^46^,^47^. Furthermore, inhibiting the JNK pathway with the inhibitor SP600125 has been reported to upregulate p53^48^,^49^. In agreement with these observations, we found that the Aβ42-induced reduction of p53 was restored by SP600125 (Fig. 3D,F). In addition to p53, we also observed that SP600125 prevented the downregulation of SIRT6 by Aβ42 (Fig. 3D,E). From these data, we suggest that the JNK pathway might be linked to the decreased expression of SIRT6 and p53 in response to Aβ42. However, further studies are required to determine whether the recovery effect of the JNK inhibitor on the Aβ42-mediated reduction of SIRT6 is p53 dependent.

The stability of p53 is mainly regulated by ubiquitin-dependent degradation, which is mediated by MDM2^50^. Nutlin-3 is a MDM2 antagonist that inhibits the interaction between p53 and MDM2 and stabilizes p53^33^. The downregulation of SIRT6 by Aβ42 was prevented when p53 levels were restored by Nutlin-3 (Fig. 4). This finding is consistent with the previous study showing that p53 positively regulates SIRT6 expression and suggests that Aβ42 decreased SIRT6 expression through MDM2-mediated degradation of p53.

DNA repair pathways are extremely important against neurodegeneration because DNA mutation rates increase with age^51^. Therefore, an understanding of the precise mechanisms and proteins involved in DNA repair is required to develop therapeutic approaches for ageing-associated diseases such as cancer and neurodegenerative disorders. We found that SIRT6 reduction perturbs the DNA repair process in the brains of an AD mouse model (Fig. 1), and we also observed that SIRT6 overexpression rescued Aβ42-induced DNA-damage (Fig. 5). The Aβ42-mediated decrease in p53 and SIRT6 plays a central role in both the DNA repair pathway and apoptosis. Genotoxic agents including ultraviolet (UV) light, ionizing irradiation (IR), and chemical agents activate and stabilize p53^52^. The role of p53 in apoptosis has been well studied, yet how p53 affects DNA repair pathways is not well-understood. The tumor suppressor p53 is an important guardian of the cellular responses to DNA damage and its activity is tightly controlled^53^,^54^. DNA damage increases the activity and protein levels of p53. It plays a protective role against DNA damage through activation of target genes involved in DNA repair such as *GADD45* and *p21*^55^,^56^. *SIRT6* was recently identified as a new target gene of p53 and is also known to regulate DNA repair^25^,^36^,^57^. We observed increased p53 levels up to 6 h following Aβ42 treatment, but its levels decreased at later time points. However, the level of γH2AX, a marker of DNA-damage, was the highest when both SIRT6 and p53 were decreased (Supplementary Figure 1). Further, we found that Nutlin-3 rescued Aβ42-induced DNA damage along with the reduction of SIRT6 protein levels (Figs 4A and 6A,C). To get insight into the link between SIRT6 and p53 on DNA repair in cells treated with Aβ42, we used SIRT6-specific siRNA and p53-deficient cell line. Even though Nutlin-3 increased the stability of p53, its protective effect against Aβ42-mediated DNA damage did not occur in HT22 cells depleted of SIRT6 by siRNA (Fig. 7). In addition, we found that Aβ42 and Nutlin-3 had no effect on SIRT6 levels and Aβ42-induced DNA damage in p53-deficient cells, repectively (Supplentary Figure 2). However, we observed that Aβ42 still increased γH2AX levels in the absence of reduction on SIRT6 because Aβ42 induces DNA damage through various pathways. Nevertheless, Nutlin-3 only prevented Aβ42-induced DNA damage when both SIRT6 and p53 exist in cells (Fig. 7 and Supplementary Figure 2). Moreover, we also observed that the effect of Aβ42 on the reduction of SIRT6 is p53-dependent using p53-deficient cell line (Supplementary Figure 3). From these data, we suggest that the effect of Nutlin-3 on Aβ42-induced DNA damage requires the upregulation of SIRT6 through p53. Since Nutlin-3 was originally developed as a non-genotoxic anti-cancer drug^33^ and is a compound that is most commonly used in anti-cancer studies^58^,^59^, our findings draw attention to new potential effects of this anti-cancer drug via p53 activation.

In summary, the present study shows for the first time three main findings. First, SIRT6 expression is decreased in the brains of both AD model mice and AD patients. Second, Aβ42 decreased the levels of SIRT6 and p53, which is related to the JNK signaling pathway. Third, SIRT6 overexpression prevented Aβ42-induced DNA damage and Nutlin-3 protected cells against Aβ42 by upregulating SIRT6. In conclusion, these findings provide a valuable insight toward the development of pharmacological SIRT6 activators for ageing-related diseases including neurodegenerative diseases, metabolic diseases and cancer. We also anticipate that SIRT6 is a novel therapeutic target for AD.

## Materials and Methods

### Cell culture and reagents

The mouse hippocampal neuronal cell line HT22 was a gifted from Dr. David Schubert (Salk Institute) and the p53-deficient human colorectal cancer cell lines (HCT116 p53^−/−^) were a gift from Dr. Kiwon Song (Yonsei University, Seoul, Korea). Cells were cultured in Dulbecco’s modified Eagle medium (DMEM) supplemented with 10% fetal bovine serum (FBS) and 0.1 mg/mL penicillin and streptomycin (P/S; Sigma-Aldrich). They were incubated in a humidified incubator at 37 °C with 5% CO_2_. Aβ_1–42_ synthetic peptide was purchased from American Peptide. SP600125 and Nutlin-3 were obtained from Sigma-Aldrich.

### Primary hippocampal neuron cultures

Primary hippocampal neurons were prepared from E18 Sprague-Dawley (SD) rat embryos, as described previously^60^ but with some modifications. The neurobasal medium to which B27 Supplement (Gibco), L-glutamine (0.5 mM), and 0.1 mg/ml penicillin and streptomycin (P/S; Sigma-Aldrich) were added was changed every 3 days. The experiments were performed in cultures at 21 days *in vitro* (DIV).

### Plasmid, siRNA and Transfection

The Flag-tagged SIRT6 expression plasmid was purchased from addgene (Cambridge, MA) (plasmid #13817). HT22 cells were transiently transfected with 1 μg/well (6 well-plates) plasmid DNA using Lipofectamine™ LTX and Plus reagent (Invitrogen) according to the manufacturer’s instruction. SIRT6 siRNA (Stealth siRNA, primer name: Sirt6MSS249886) was pre-designed and synthesized by Life Technologies (Life Technologies Corporation) and HT22 cells were transfected with SIRT6 siRNA using RNAiMAX reagent (Invitrogen).

### Western Blot

After treatment, cells were harvested using RIPA buffer with protease/phosphatase inhibitors. The samples were then sonicated for 3 s and centrifuged at 13,000 rpm for 15 min at 4 °C. The supernatant was collected as whole cell extracts and protein samples were loaded on NuPAGE 4–12% Bis-Tris gel (Novex Life technologies) and transferred to PVDF membranes. The membrane was incubated overnight at 4 °C with specific antibodies, such as Anti-SIRT6, anti-γH2AX, anti-acH3K56, anti-acH3K9, and anti-Histone H3 that were obtained from Abcam. Anti-β-actin and anti-p53 were purchased from Sigma-Aldrich and Cell signaling, respectively. The bands were detected using the image analyzer LAS-3000 (Fujifilm) using a western blot detecting kit (Youngin frontier). Band intensities were quantified with Fujifilm Multi Gauge 3.0 Software.

### RNA Preparation and Real-time PCR

Total RNA was isolated using the Qiagen RNeasy kit (Qiagen) and cDNA was synthesized as described previously. Quantitative real-time PCR was performed using an ABI stepone 2.1 (Applied Biosystems, Foster City, CA, USA). The primers used for mouse SIRT6 were: 5′-GGCTACGTGGATGAGGTGAT-3′ (forward) and 5′-GGCTCAGCCTTGAGTGCTAC-3′ (reverse); for p53: 5′- TGAAACGCCGACCTATCCTTA-3′ (forward) and 5′-GGCACAAACACGAACCTCAAA-3′ (reverse); and for GAPDH: 5′- CATGGCCTTCCGTGTTCCTA-3′ (forward) and 5′- CCTGCTTCACCACCTTCTTGAT-3′ (reverse). Relative mRNA expression of target genes was normalized to the endogenous GAPDH control gene. The primers used for human SIRT6 were: 5′-CATCCTAGACTGGGAGGA-3′ (forward) and 5′-CAGGTTGACGATGACCAG-3′ (reverse).

### Immunofluorescence assays

For immunofluorescence, the cells were fixed in 4% paraformaldehyde and permeabilized with 0.1% Triton X-100. Cells were washed with phosphate-buffered saline (PBS) and blocked with PBS containing 5% bovine serum albumin (BSA), and incubated with primary antibodies overnight at 4 °C. Primary antibodies were used at the following dilutions: SIRT6 (abcam; 1:500) and γH2AX (abcam; 1:500). Following three washes in PBS, cells were incubated with Alexa Fluor-conjugated secondary antibodies for 1 h at room temperature. The cells were then washed and labeled with DAPI. The images were acquired using a laser scanning confocal microscope (LSCM; Olympus Fluoview 300). The fluorescence intensities of SIRT6 were analyzed using Image J software (NIH, Bethesda, MD, USA) and the number of γH2AX foci in >70 cells in each treatment condition was counted randomly.

### Alkaline Comet Assay

Comet assays were performed using a comet assay kit (catalog number; 4250-050-K, Trevigen) according to manufacturer’s instruction. DNA was labeled with SYBR Green dye. The images were taken by a fluorescence microscope (Olympus, Tokyo, Japan). The tail moment (tail DNA% × length of tail) was analyzed automatically from at least 100 cells per each sample with Comet Assay IV (ver 4.3.2) software (Perceptive Instruments). The length of tail was measured from the center of the head to the center of the tail.

### Animals

Alzheimer’s disease model mice, 5XFAD (The Jackson Laboratory, Bar Harbor, ME; stock no. 006554, Tg6799), were used for western blot and DAB staining and maintained in Seoul National University’s mouse facility. The mice express the human amyloid precursor protein bearing the Swedish (K670N, M671L), Florida (I716V), and London (V717I) mutations and two human presenilin-1 mutations (M146L, L286V). All animal experiments were performed in accordance with the Principle of Laboratory Animal Care (NIH publication No. 85–23, revised 1985) and the Animal Care and Use Guidelines of Seoul National University, Seoul, Korea. All experimental protocols were approved by Institutional Animal Care and Use Committee (IACUC) in Seoul National University Hospital.

### Human brain samples

Neuropathological processing of normal and AD human brain samples was performed using procedures previously established by the Boston University Alzheimer’s Disease Center (BUADC). All brains were donated with consent of the next of kin after death. Institutional review board approval was obtained through the BUADC. The study was performed in accordance with institutional regulatory guidelines and principles of human subject protection in the Declaration of Helsinki. Detailed information of the brain tissues is described in Table 1 in Supplementary information.

### Immunohistochemistry

Immunohistochemistry for histological analysis, the free-floating sections were pretreated with 1% hydrogen peroxide for 20 min to quench endogenous peroxidase activity. Tissue sections were incubated overnight with mouse anti-SIRT6 antibody (1:100, Thermo scientific). The sections were incubated with biotinylated-mouse secondary antibody (1:200, Vector Laboratories, Burlingame, CA) and then visualized using the avidin-biotin-peroxidase complex (ABC) method with 3,3 -diaminobenzidine tetrahydrochloride (DAB) as the chromogen. The sections were mounted on glass slides, air-dried, dehydrated by subjecting to an increasing-concentration alcohol series, cleared in xylene, and cover-slipped with Permount (Fisher Scientific). Immunohistochemistry images were taken under a fluorescence microscope (IX71, Olympus). To analyze SIRT6 staining, the number of DAB-positive cells from the captured images was counted using the Image J program (NIH, Bethesda, MD, USA).

### Statistical analysis

Data are presented as mean ± standard error of the mean (SEM). The t-test or one-way ANOVA with Tukey’s multiple comparison tests was used to analyze the statistical significance of the results using the GraphPad Prism 5 software. P < 0.05 was accepted as a statistically significant difference.

## Additional Information

**How to cite this article**: Jung, E.S. *et al*. p53-dependent SIRT6 expression protects Aβ42-induced DNA damage. *Sci. Rep*. **6**, 25628; doi: 10.1038/srep25628 (2016).

## Supplementary Material

Supplementary Information
