# Supplementary material for: p53-dependent SIRT6 expression protects Aβ42-induced DNA damage
Source: Sci Rep. 2016 May 9;6:25628. doi: 10.1038/srep25628 (PMC4860716; doi:10.1038/srep25628)

Supplementary Information

p53-dependent SIRT6 expression protects Aβ42-induced DNA damage

Eun Sun Jung[1], *Hyunjung Choi*[1], *Hyundong Song*[1], *Yu Jin Hwang*[2], *Ahbin Kim*[1], *Hoon Ryu*[2,3], *Inhee Mook-Jung*[1*]

*Corresponding Author:

Inhee Mook-Jung, PhD
Department of Biochemistry and Biomedical Sciences, Seoul National University, College of Medicine, 103 Daehak-ro, Jongro-gu, Seoul 03080, Korea
E-mail) inhee@snu.ac.kr
Phone number) +82-2-740-8245
Fax number) +82-2-3672-7352

Supplementary figure 1.

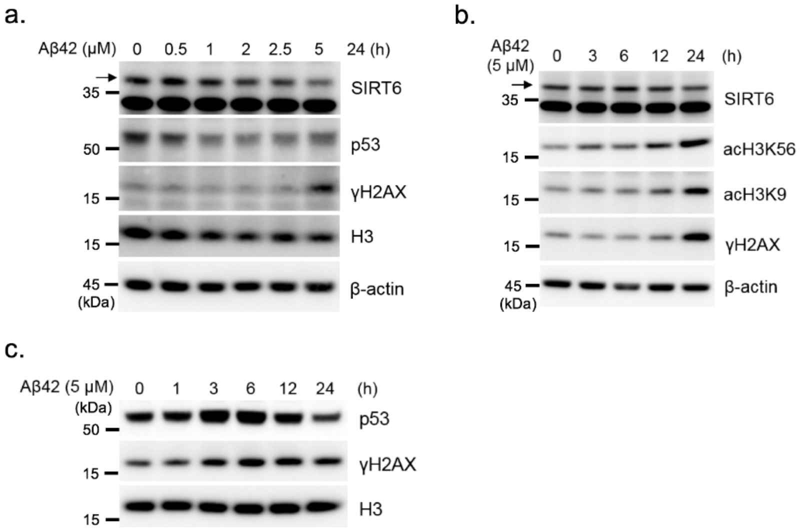

Figure S1. Aβ42 affects the level of SIRT6, acH3K9, acH3K56, p53 and γH2AX.
a-c. HT22 cells were treated with indicated concentration of Aβ42 for 24h and treated with 5 μM Aβ42 for different time points. Cells were harvested for immunoblotting with anti-SIRT6, anti-p53, anti-γH2AX, anti-acH3K9, anti-acH3K56, anti-histone H3 and anti-β-actin. β-actin and histone H3 were used as loading control. The arrow indicates the SIRT6 band.

Supplementary figure 2.

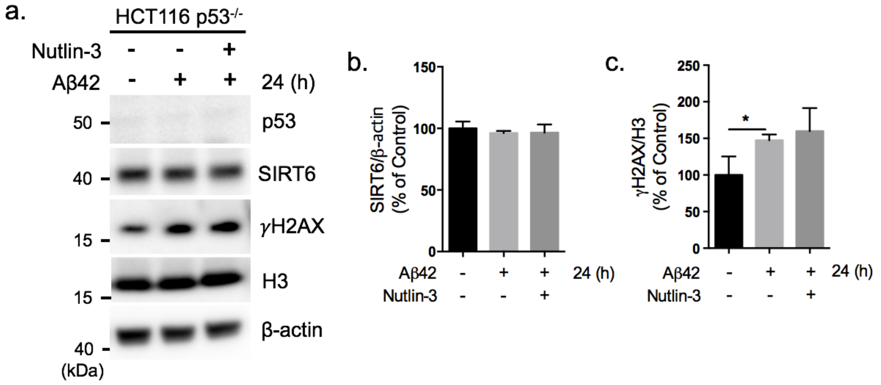

Figure S2. Aβ42 does not decrease the level of SIRT6 and Nutlin-3 has no effect on Aβ42-induced DNA damage in p53 deficient cells.
a-c. HCT116 p53-deficient cells (HCT116 $p53^{-/-}$) were treated with Aβ42 (5 μM) for 24 h in the absence or in the presence of Nutlin-3. **a.** Representative image of western blotting. Cells were harvested for immunoblotting with anti-p53, anti-SIRT6, anti-γH2AX, anti-histone H3 and anti-β-actin. β-actin and histone H3 were used as loading control. **b, c.** Quantification of SIRT6 and γH2AX (n=4, *P < 0.05, unpaired t-test).

Supplementary figure 3

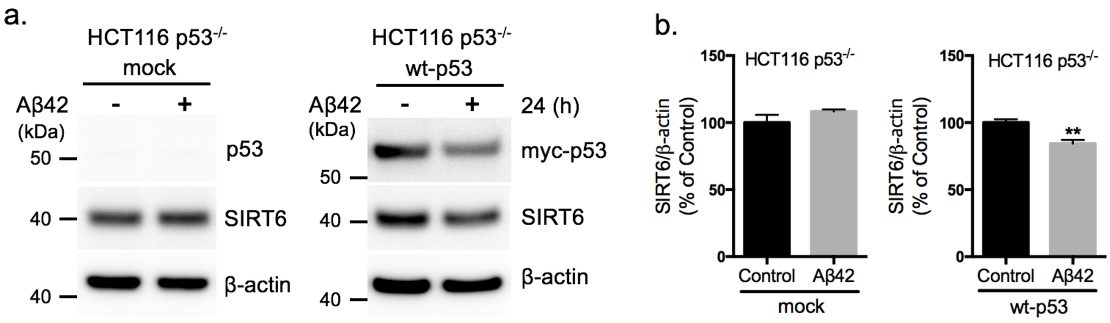

Figure S3. Aβ42-induced reduction of SIRT6 levels is p53-dependent.
HCT116 p53-/- cells were transfected with mock vector (pcDNA3.1) or myc-tagged wild-type p53 vector and then treated with Aβ42 for 24 hours. The cell lysates were immunoblotted with indicated antibodies. **a.** Representative image of western blotting. **b.** Quantification of SIRT6 (n=4, **$P < 0.01$, unpaired t-test).

Supplementary Table 1.

Human brain tissue information for normal subjects and AD patients. In all cases where AD was diagnosed at autopsy, AD was stated as the cause of death.

Case	Age	Sex	Braak stage	Cause of Death
**Normal 1**	87	F	I	Unknown
**Normal 2**	86	M	II	Unknown
**Normal 3**	67	M	I	Sudden accidental death
**Normal 4**	101	F	I	Myocardial infarction
**Normal 5**	68	M	I	Congestive heart failure
**Normal 6**	78	F	I	Unknown
**AD 1**	82	M	V	AD
**AD 2**	79	F	VI	AD
**AD 3**	100	M	V	AD
**AD 4**	75	M	V	AD
**AD 5**	83	M	VI	AD
**AD 6**	79	F	VI	AD

Full-length Western blot images

Figure 1A

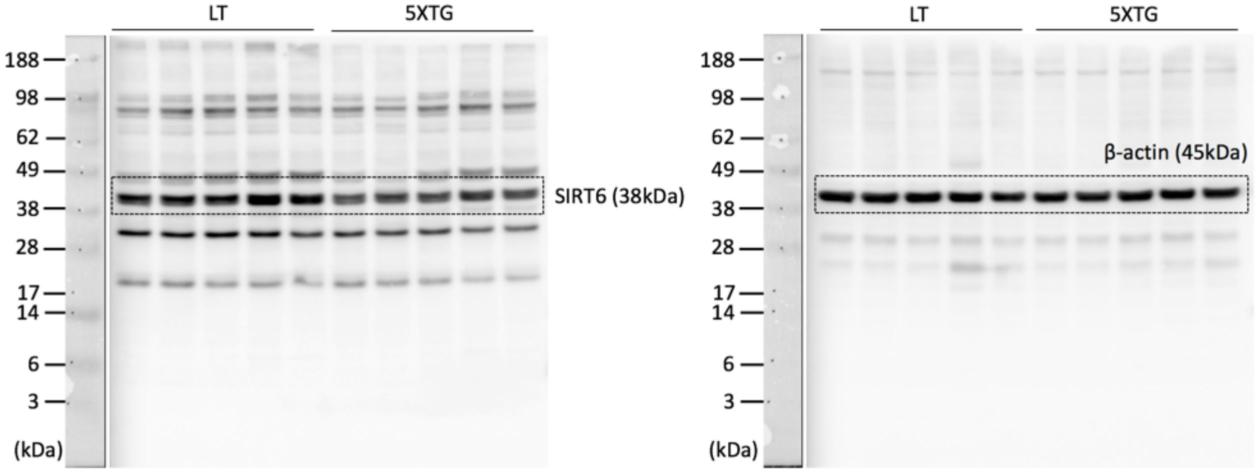

Figure 1F

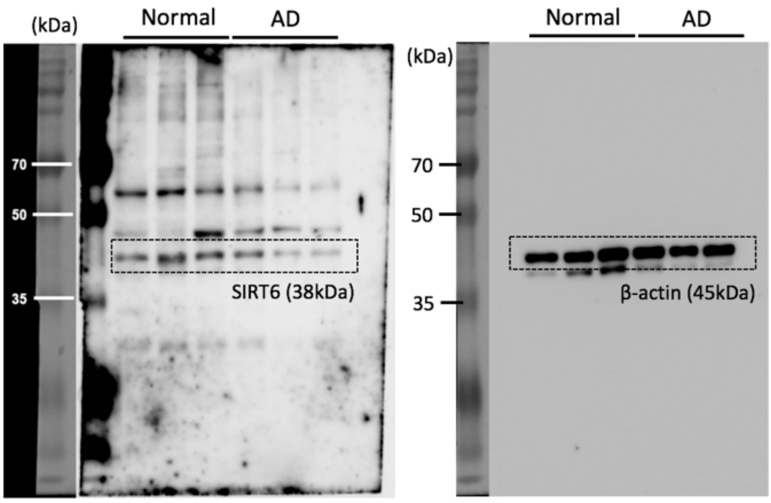

Figure 2A

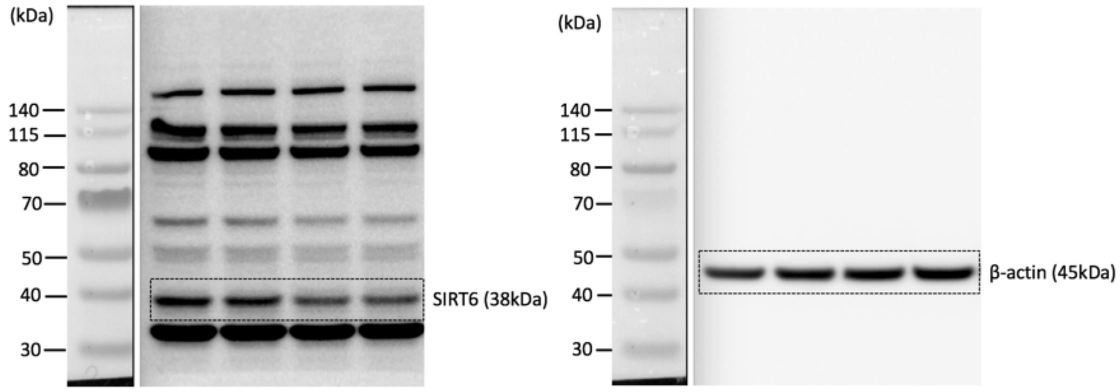

Figure 2E

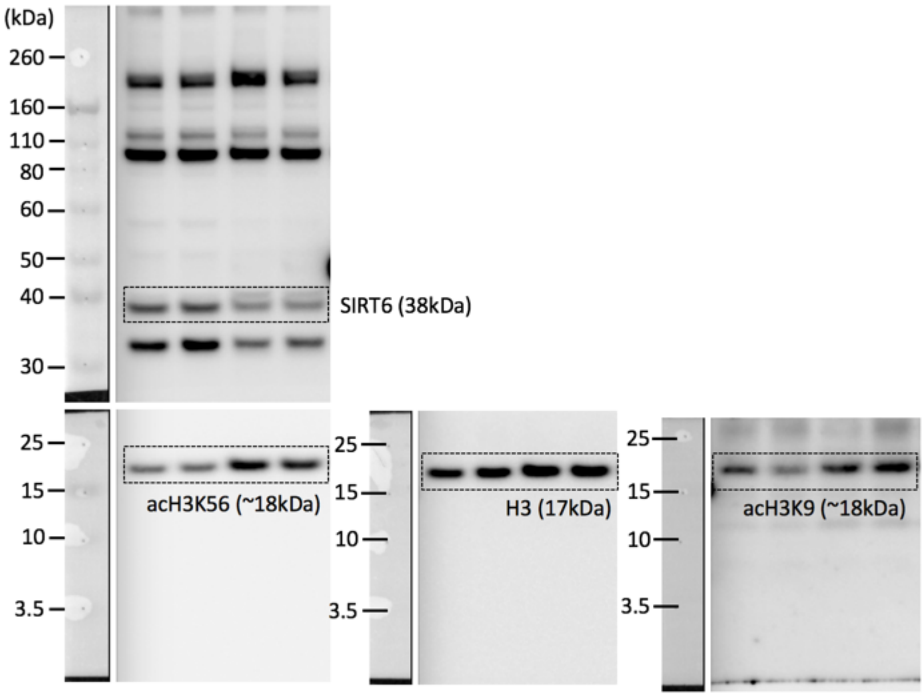

Figure 2G

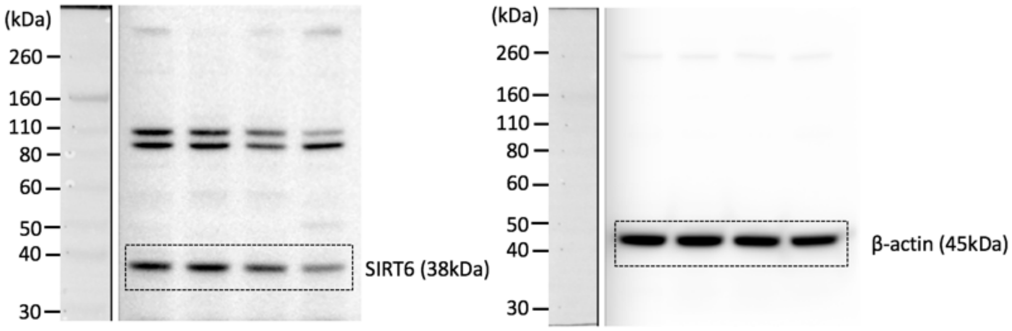

Figure 3A

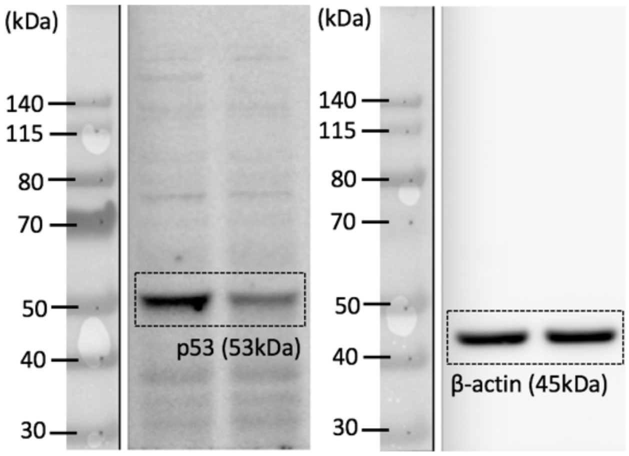

Figure 3D

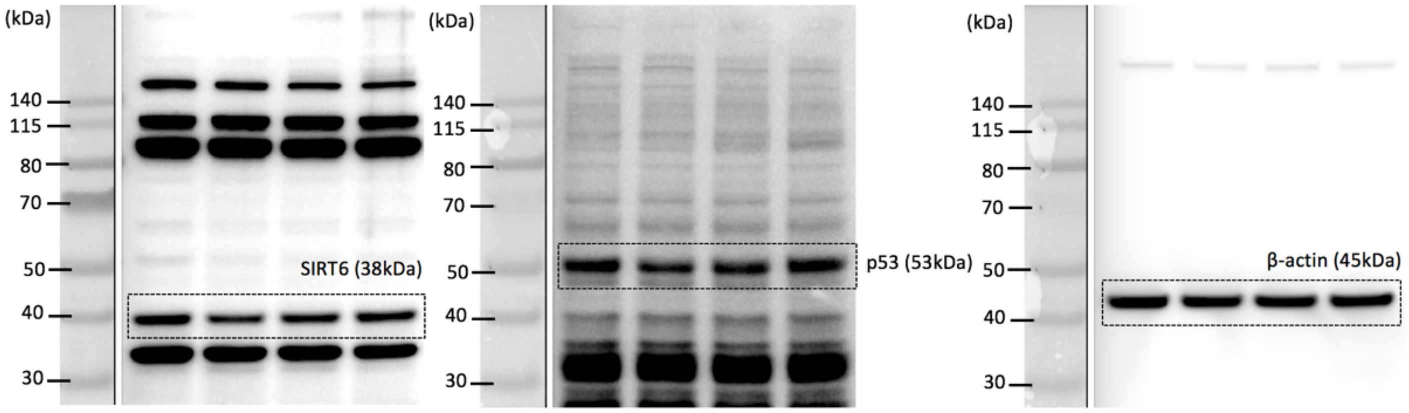

Figure 4A

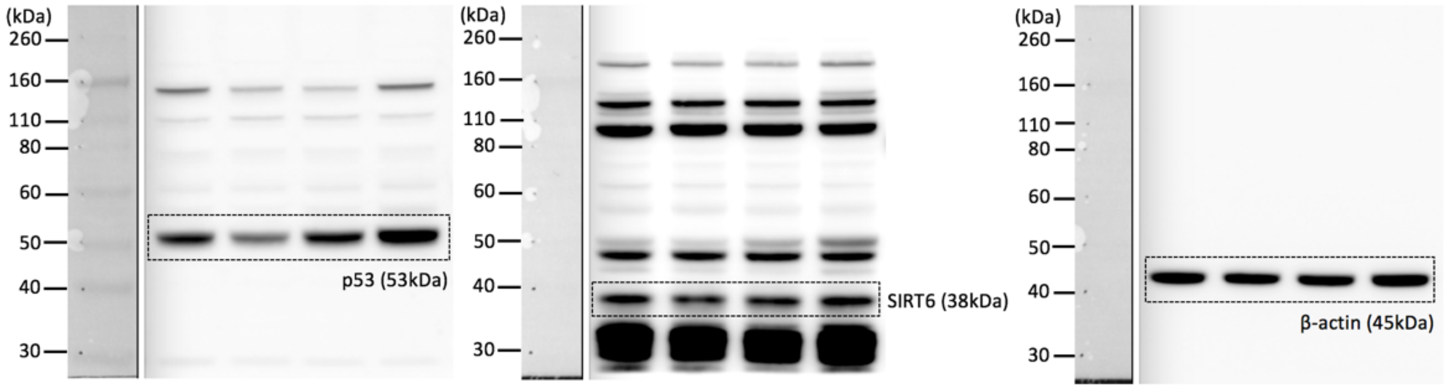

Figure 4F

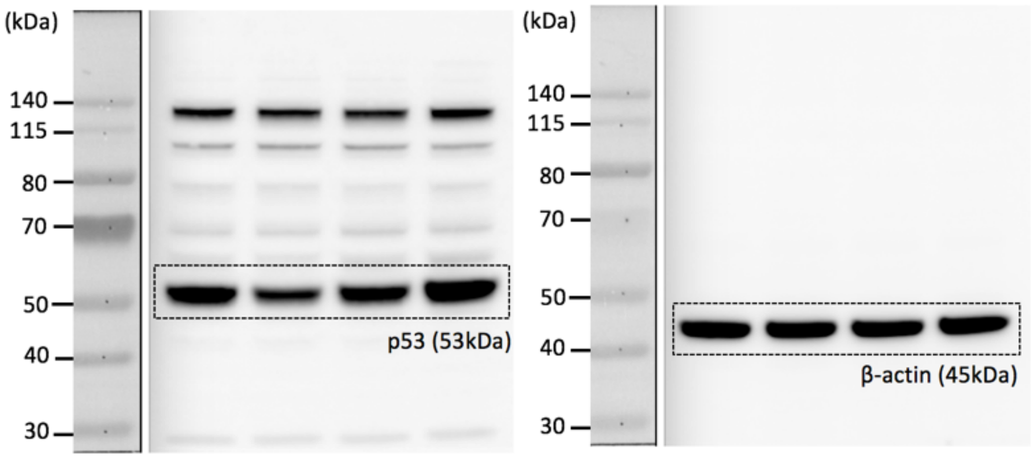

Figure 5A

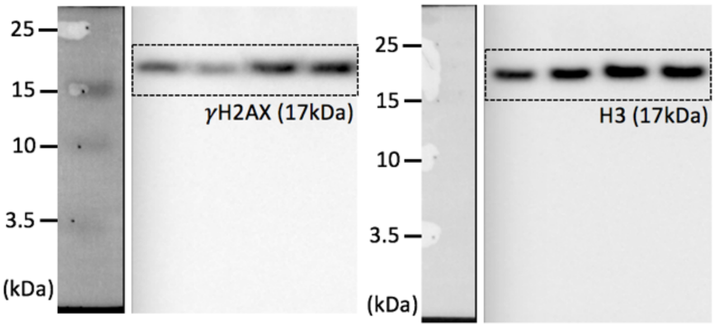

Figure 5E

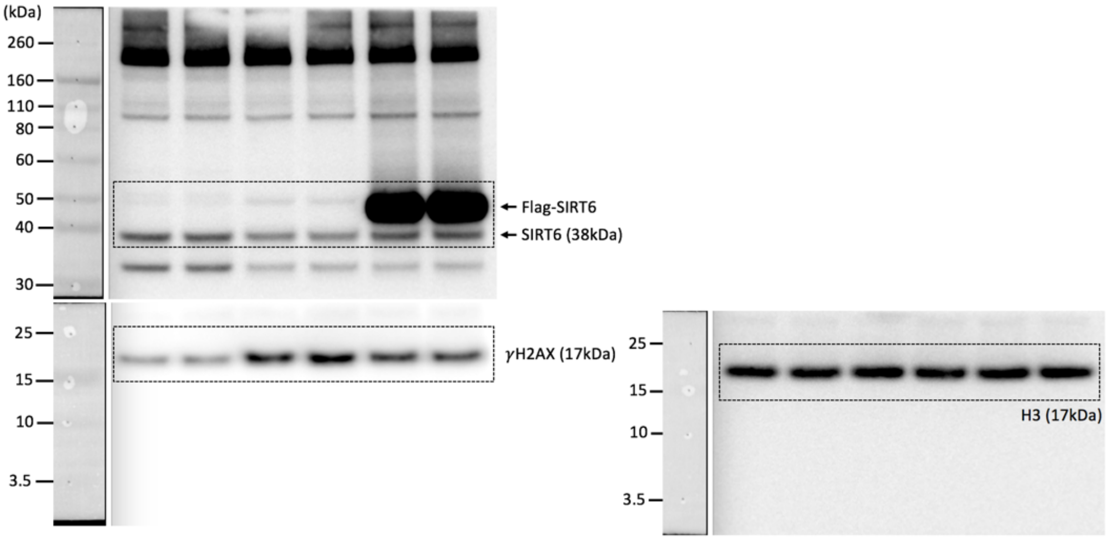

Figure 5I

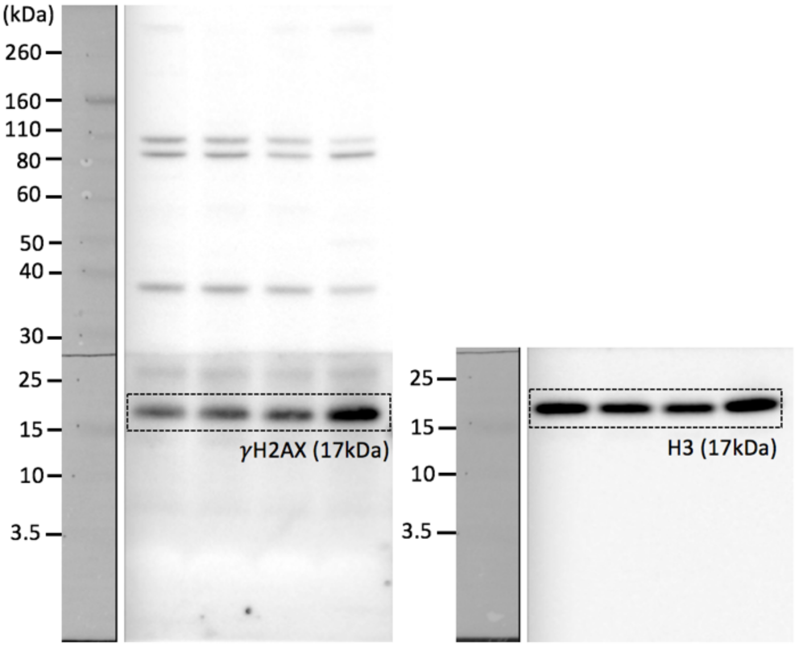

Figure 6C

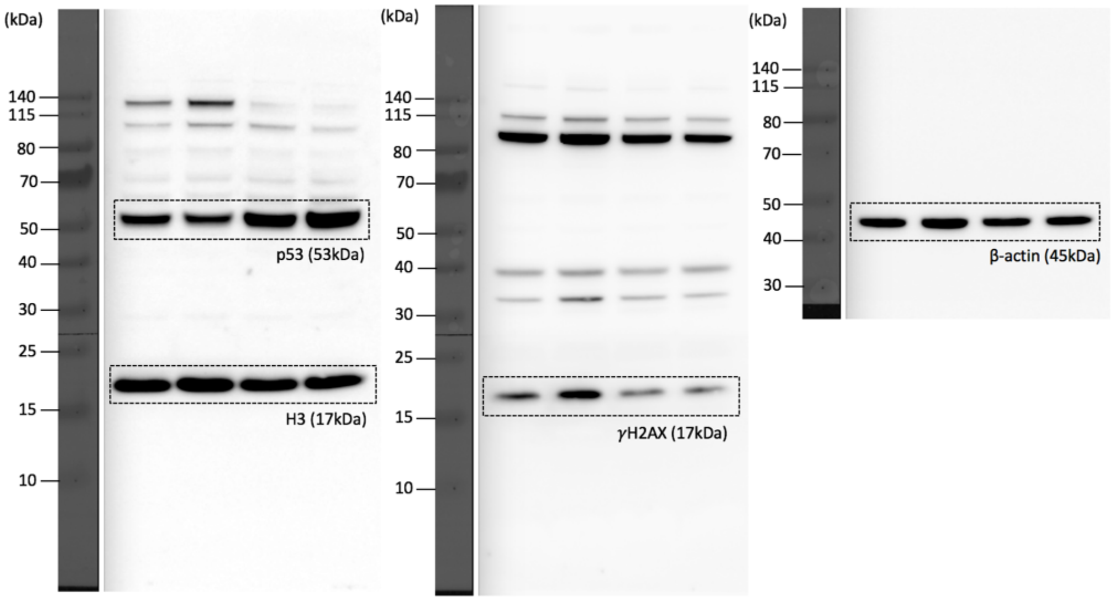

Figure 7A

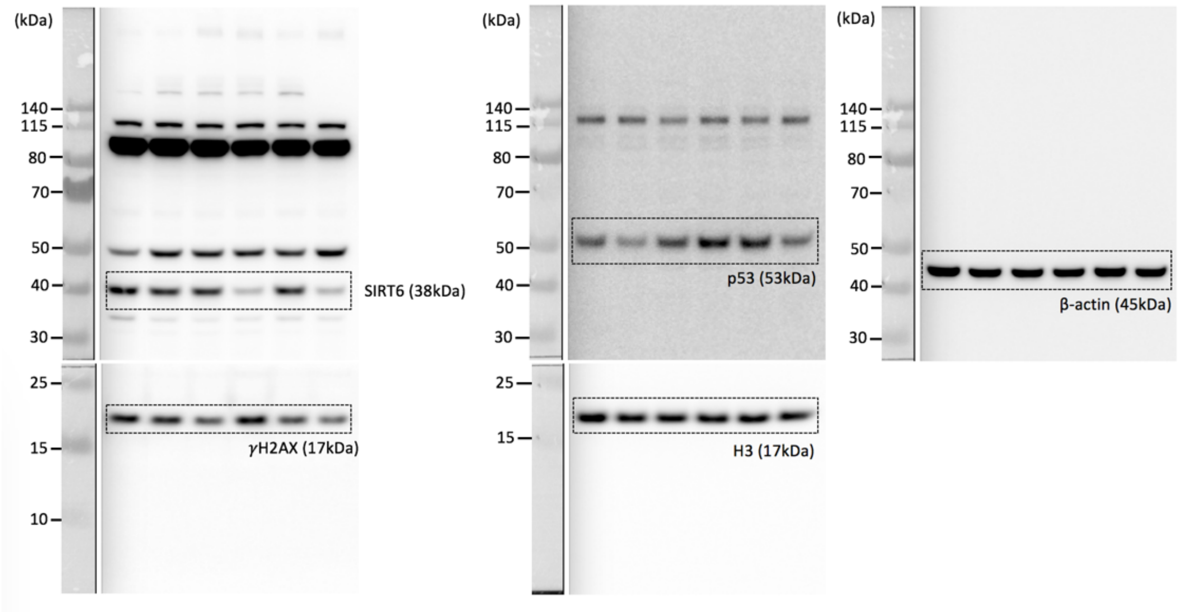

Supplement: Supplementary Information [file srep25628-s1.pdf]
